# Value‐Based Neuromonitoring in Thyroidectomy: A Comprehensive Cost–Utility Analysis

**DOI:** 10.1002/lary.70536

**Published:** 2026-03-31

**Authors:** Daqi Zhang, Francesco Brucchi, Carla Colombo, Roberto Cirocchi, Gianlorenzo Dionigi

**Affiliations:** ^1^ Division of Thyroid Surgery China‐Japan Union Hospital of Jilin University, Jilin Provincial Key Laboratory of Thyroid Disease, Jilin Provincial Precision Medicine Laboratory of Molecular Biology and Translational Medicine on Differentiated Thyroid Carcinoma Changchun Jilin China; ^2^ Division of General Surgery Istituto di Ricovero e Cura a Carattere Scientifico (IRCCS) Istituto Auxologico Italiano Milan Italy; ^3^ Department of Pathophysiology and Transplantation University of Milan Milan Italy; ^4^ Division of Endocrinology Istituto di Ricovero e Cura a Carattere Scientifico (IRCCS) Istituto Auxologico Italiano Milan Italy; ^5^ Department of Medicine and Surgery, S. Maria Hospital University of Perugia Terni Italy

**Keywords:** continuous intraoperative neuromonitoring (CIONM), cost‐effectiveness, cost–utility, health economics, intraoperative neuromonitoring (IONM), nerve trend monitoring/automated EMG trend monitoring, postoperative laryngoscopy, recurrent laryngeal nerve injury, technical failure, thyroidectomy

## Abstract

**Objective:**

To compare the cost–utility of major intraoperative neuromonitoring (IONM) strategies in thyroidectomy across commercial platforms, and to quantify the impact of continuous IONM (CIONM), automated nerve trend monitoring, postoperative laryngoscopy policy, and technical failure on total episode costs and medicolegal burden.

**Methods:**

A decision‐analytic cost–utility model of 6000 thyroidectomies was developed using full pathway micro‐costing and QALY outcomes. Strategies included visual identification, intermittent IONM, CIONM, needle‐based IONM, and automated EMG trend monitoring, evaluated in low‐ and high‐utilization settings and stratified by manufacturer. The model incorporated RLN injury costs, routine versus EMG‐guided selective laryngoscopy, and modality‐specific technical failure rates.

**Results:**

Higher neuromonitoring utilization substantially reduced monitoring costs (€420 to €260 per case), with routine CIONM achieving the lowest values (~€240). Intermittent IONM and CIONM lowered permanent/transient RLN palsy rates from 2.0%/6.0% (visual identification) to 1.2%/4.0% and 0.8%/3.0%, reducing RLN‐related costs from €650 to €430 and €310 per case. Total per‐case costs were €4400 (no monitoring), €4250 (intermittent), and €4100 (CIONM), with CIONM remaining cost‐effective (ICER ≈€8000). Selective EMG‐guided laryngoscopy reduced laryngoscopy costs by up to 65% and improved ICERs. Technical failures increased costs by €90–€110 per case but were mitigable. Manufacturer analyses showed similar per‐case costs (€250–€310) in high‐utilization settings.

**Conclusion:**

Routine CIONM in high‐volume centres is the most economically favorable strategy, yielding substantial savings and ICERs within accepted thresholds. Selective EMG‐guided laryngoscopy and minimizing technical failures further enhance cost–utility. Automated trend‐monitoring platforms offer a pragmatic, near‐equivalent alternative where full CIONM deployment is limited.

**Level of Evidence:**

5.

## 1 Introduction

Intraoperative neural monitoring (IONM)—including intermittent IONM, continuous IONM (CIONM), needle‐based IONM, and automated nerve trend analysis—has become a core adjunct to modern thyroid surgery [1, 2, 3, 4]. Its principal clinical rationale is to reduce recurrent laryngeal nerve (RLN) and external branch of the superior laryngeal nerve (EBSLN) palsy and to mitigate bilateral RLN paralysis through staged bilateral procedures [5, 6, 7, 8].

Over the past decade, an expanding range of commercial neuromonitoring platforms has created a heterogeneous technological landscape, with variability in capital investment, disposable use, maintenance contracts, and training requirements (see Table S1) [9]. This diffusion of technology has preceded structured health technology assessment and formal cost‐effectiveness appraisal, challenging the principles of value‐based surgical innovation [10, 11, 12, 13, 14]. From an economic perspective, the additional intraoperative costs associated with IONM—including equipment purchase, consumables, staff time, and theater overheads—must be weighed against downstream costs related to nerve injury, rehabilitation, medicolegal claims, productivity losses, and long‐term healthcare utilization [13, 14].

Most published evaluations have focused on RLN injury rates and short‐term functional outcomes, often within single devices or limited clinical scenarios, and have rarely incorporated a full cost‐utility framework with quality‐adjusted life years (QALYs) and payer‐level budget impact [15, 16, 17, 18, 19, 20].

The present study develops a device‐level, decision‐analytic cost–utility model that systematically compares all major commercially available IONM and CIONM systems used in thyroid surgery. By quantifying incremental costs, QALYs, and budget impact across monitoring strategies and practice volumes, this work aims to provide a rigorous health economic framework to support evidence‐based technology assessment, rational resource allocation, and sustainable implementation of neuromonitoring in endocrine surgery.

## 2 Methods

### 2.1 Study Design

This economic evaluation used a decision‐analytic cost–utility simulation [21, 22] adopting hospital and regional payer perspectives and a micro‐costing framework to estimate direct and indirect costs related to RLN injury and its prevention [21]. A decision‐tree model incorporated health states for no RLN injury, temporary palsy, and permanent palsy, with transition probabilities and utilities derived from institutional data and published sources [15, 16, 17, 18, 19, 20]. Cost and QALY estimates spanned the index operation to the long‐term consequences of RLN injury, using a lifetime horizon for quality‐of‐life effects and shorter horizons for selected cost components, with values expressed in current‐year Euros and discounted per national guidelines [21, 22]. Strategies compared included visual identification, laryngeal palpation, needle‐based IONM, intermittent IONM, CIONM, and automated EMG trend monitoring across major manufacturers [1, 2, 3, 4, 23, 24]. The protocol was approved by the local ethics committee (ME2024‐1644) and conducted in accordance with the Declaration of Helsinki.

### 2.2 Patient Pathway Analysis

Direct medical costs across the full thyroidectomy continuum were estimated using activity‐based, bottom‐up micro‐costing (Data S1 and S2). The pathway was divided into preoperative, intraoperative, and postoperative phases, each associated with specific cost drivers such as personnel time, consumables, device use, diagnostics, and overheads [21, 22, 25, 26, 27, 28]. Preoperative assessments included consultations, laryngoscopy, laboratory tests, and imaging; intraoperative costs reflected operative and anesthetic time, theater occupancy, neuromonitoring setup and troubleshooting, and device‐related disposables; postoperative costs covered recovery, ward stay, analgesia, calcium and vocal monitoring, early complication management, and initial follow‐up [23, 29]. Resource use was triangulated from administrative databases, theater logs, and structured staff interviews. Distinct care pathways were constructed for each monitoring strategy to capture differences in setup, consumables, troubleshooting, and downstream RLN‐related management, enabling comparative costing and incremental cost–utility analysis [21, 22].

### 2.3 Economic Evaluation and Costing

Unit costs were derived from hospital cost‐accounting systems, regional reimbursement schedules, and procurement price lists (Data S2). Capital costs for neuromonitoring consoles were annualized and allocated per case based on hospital‐ and surgeon‐level procedure volume, distinguishing predefined high‐ and low‐volume operators [30]. Device‐specific costs—including purchase, maintenance, disposables (EMG tubes, vagal or needle electrodes, stimulators), and training or software upgrades—were quantified for each manufacturer [9]. Indirect RLN‐related costs (rehabilitation, long‐term speech therapy, medicolegal expenses, productivity losses) were estimated from contemporary literature and local data [15, 16, 17, 18, 19, 20]. Costs were stratified by monitoring modality (visual identification, laryngeal palpation, intermittent IONM, CIONM, automated trend monitoring) and by recording configuration (integrated EMG tube vs. stitched or surface electrodes), reflecting current practice [1, 2, 3, 4, 23]. Capital and maintenance costs for neuromonitoring consoles were annualized and allocated per case based on center and surgeon procedure volumes and modality‐specific utilization rates, so that high‐volume, routine CIONM programs amortized fixed expenses over a larger monitored caseload than selectively used intermittent IONM. This utilization‐dependent capital allocation, combined with modality‐specific disposable configurations (integrated EMG tube versus stitched, surface, or needle electrodes), explains why routine CIONM could have a lower per‐case monitoring cost than selectively deployed intermittent IONM, despite requiring an additional vagal electrode.

### 2.4 Study Arms and Subgroup Analyses

Study arms included (see Data S3): (1) no neuromonitoring with visual identification alone; (2) laryngeal palpation to assess nerve integrity [24]; (3) needle‐based IONM with percutaneous or transcartilaginous electrodes [3]; (4) intermittent IONM using standardized stimulation protocols [23]; (5) continuous IONM (CIONM) with real‐time vagal or RLN surveillance [7]; and (6) automated nerve trend–monitoring platforms with continuous EMG analysis and alarm thresholds [4]. Subgroup analyses examined electrode configuration (integrated EMG tube vs. stitched or surface electrodes) [23], surgical volume at surgeon and hospital level (high vs. low) [30], and proportional RLN risk reduction derived from pooled contemporary series [15, 16, 17, 18, 19, 20].

### 2.5 Outcomes

Primary outcomes were the total per‐patient cost of neuromonitoring (capital, disposables, maintenance, training) and the net cost offset derived from differences in RLN injury–related costs between monitored and unmonitored strategies. Secondary outcomes included the use and cost impact of routine versus selective laryngoscopy, direct and indirect per‐patient costs (hospitalization, rehabilitation, medicolegal and productivity losses), QALYs gained or lost based on state‐specific utilities, and the incidence and cost consequences of technical failures. Incremental cost–effectiveness ratios (ICERs) were calculated by comparing each strategy with visual identification alone and then with the next most effective non‐dominated option, expressed as cost per RLN injury averted and per QALY gained [31].

### 2.6 Model Inputs and Assumptions

Utilities for each health state (no RLN injury, temporary RLN palsy, permanent RLN palsy, and, where applicable, more severe outcomes such as tracheostomy or bilateral RLN injury) were sourced from the literature on thyroid surgery and voice‐related quality of life, and mapped to QALYs over the chosen time horizon [15, 16, 17, 18, 19, 20]. These proportional reductions were applied consistently across study arms, with uncertainty characterized through predefined ranges and probability distributions. Assumptions regarding the frequency of staged bilateral surgery, re‐interventions, and long‐term specialist follow‐up for patients with RLN injury were explicitly incorporated into the model. All parameter values, ranges, and data sources are reported in Data S1 and S2, together with justification of key structural assumptions. Baseline probabilities of transient and permanent RLN palsy under visual identification alone and under each monitoring modality were derived from contemporary meta‐analyses and large multicenter series, representing mid‐range values across a spectrum of surgeons and institutions rather than the lowest rates achievable by expert high‐volume operators. Scenario and sensitivity analyses also evaluated ‘high‐volume, low‐injury’ conditions by proportionally reducing absolute palsy probabilities (e.g., toward 1.0%, 0.6%, and 0.4% permanent palsy for no monitoring, intermittent IONM, and CIONM, respectively) while preserving observed relative risk reductions.

### 2.7 Definition of Monitoring Technical Failure

IONM “technical failure” was defined as any intraoperative neuromonitoring malfunction that prevented reliable RLN assessment despite attempted troubleshooting, in the absence of true nerve injury [15, 16, 17, 18, 19, 20, 23]. Failure modes included in the simulation comprised: blown fuses or console power faults, fractured or short‐circuited EMG endotracheal tube electrodes, inappropriate anesthetic techniques (e.g., neuromuscular blockade, inadequate depth), defective or unavailable stimulators, lack of compatible replacement disposables, and CIONM‐specific problems such as dislocation or non‐deployability of vagal electrodes [15, 16, 17, 18, 19, 20, 23]. Based on published series reporting device‐related loss of signal and equipment malfunction, the base‐case analysis assumed an overall technical failure rate of 4% for intermittent IONM and 6% for CIONM, with higher rates during early implementation phases and in low‐volume centres [15, 16, 17, 18, 19, 20, 23]. Base case technical failure rates were set at 4% for intermittent IONM and 6% for CIONM, reflecting pooled estimates from heterogeneous series that include early implementation phases, low‐volume centers, and a broad spectrum of device‐related loss of signal events [15, 16, 17, 18, 19, 20, 23]. To account for experienced, high‐volume programs, deterministic and probabilistic sensitivity analyses explored substantially lower failure frequencies, down to values approximating 1% for intermittent IONM, confirming that the relative ranking of monitoring strategies remained unchanged across plausible ranges.

### 2.8 Manufacturer‐Specific Analyses

Manufacturer‐specific analyses focused on the main multipurpose neuromonitoring platforms currently used in thyroid and parathyroid surgery (see Table S1). For each platform, the model incorporated monitoring modality (intermittent versus continuous), recording configuration (EMG endotracheal tube, vagal electrodes, needle electrodes), and integration within standardized IONM algorithms for RLN protection. Manufacturer‐level heterogeneity was thus reflected in both cost inputs and, where evidence permitted, in performance parameters, while preserving a common structural framework across all study arms.

### 2.9 Statistical and Comparative Analysis

Statistical and comparative analyses combined conventional biostatistics with economic modeling. Categorical outcomes (temporary and permanent RLN injury) were compared using chi‐square or Fisher's exact tests, and continuous outcomes (cost per case, operative time) using ANOVA, linear regression, or generalized linear models for skewed data, with non‐parametric tests as needed. Multivariable logistic regression generated adjusted odds ratios and 95% CIs for associations between monitoring strategy and RLN injury, informing baseline probabilities and relative risk reductions in the decision model [31]. Economic evaluation followed standard cost‐effectiveness methods, calculating ICERs (Δcost/Δeffect) based on QALYs gained and permanent palsies averted. Uncertainty was assessed through deterministic one‐way and multi‐way sensitivity analyses and probabilistic Monte Carlo simulation using parametric distributions for probabilities, costs, utilities, and relative risks, producing cost‐effectiveness planes, acceptability curves, and incremental net monetary benefit estimates [32].

### 2.10 DRG‐Based Comparison and Budget Impact

Total hospitalization and procedure costs in each scenario were compared with the corresponding reimbursement tariff under the Italian DRG 290 code for thyroid surgery [33]. These comparisons were stratified by monitoring modality and by volume category at both hospital and surgeon levels.

### 2.11 Justification of the Simulated 6000 Procedure Cohort

The decision‐analytic model was populated with a simulated cohort of 6000 thyroidectomy procedures. This cohort size was selected to approximate the total annual volume of thyroid surgery across low‐ and high‐volume centres in the reference health system, and to ensure adequate representation of different neuromonitoring adoption patterns (selective versus routine IONM/CIONM) and practice settings [34, 35, 36]. By distributing fixed capital, maintenance and training costs over 6000 cases, the model captures utilization‐dependent economies of scale and provides stable estimates of average monitoring cost per case within each scenario, while minimizing stochastic variability in cost and effect outputs [34, 35, 36]. The simulated cohort included a realistic mix of total thyroidectomies and lobectomies, reflecting current endocrine surgery practice in the reference health system. Proportions were based on institutional administrative data and published series. The model explicitly incorporated staged bilateral thyroidectomy, with the probability of conversion from planned single‐stage to two‐stage surgery, as well as the additional operating room, anesthesia, hospitalization, and RLN‐related costs of the second stage, parameterized separately for each monitoring strategy.

## 3 Results

### 3.1 Utilization of Neuromonitoring and Monitoring‐Related Costs

**FIGURE 1:**
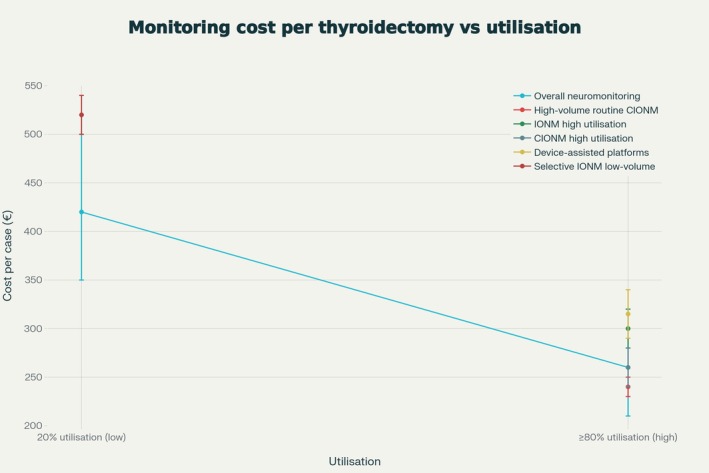
Monitoring cost per thyroidectomy versus neuromonitoring utilization and modality. The figure shows the relationship between neuromonitoring utilization and monitoring cost per thyroidectomy case. On the x‐axis, two utilization scenarios are presented: low utilization (IONM/CIONM in 20% of procedures) and high utilization (monitoring in ≥ 80% of procedures). On the y‐axis, mean monitoring cost per case (in euros) is plotted, with vertical error bars indicating 95% uncertainty intervals or plausible cost ranges. The “Overall neuromonitoring” series shows a marked decrease in cost per case from low to high utilization, reflecting capital amortization and economies of scale. Additional series display modality‐specific costs at high utilization: intermittent IONM, continuous IONM (CIONM), and device‐assisted platforms with automated EMG analysis, as well as selective IONM in low‐volume centres at low utilization, which shows the highest per‐case cost and highlights the penalty of under‐utilization of installed systems. [Color figure can be viewed in the online issue, which is available at www.laryngoscope.com]

Across 6000 simulated thyroidectomies, greater neuromonitoring utilization progressively reduced per‐case monitoring costs, from €420 at 20% use to €260 at ≥ 80% (−38%) (Figure 1). High‐volume centres using routine CIONM achieved the lowest costs (~€240) through optimal capital amortization. At high utilization, intermittent IONM, CIONM, and automated trend monitoring cost €280–€320, €240–€280, and €290–€340 per case, respectively, whereas selective low‐volume use exceeded €500, highlighting the cost penalty of under‐utilized platforms. In these high utilization scenarios, lower per‐case monitoring costs for routine CIONM compared with selectively used intermittent IONM were due to more favorable amortization of capital and maintenance expenses and, in some configurations, to the use of lower‐cost EMG recording options (stitched or surface electrodes) rather than intrinsically cheaper CIONM disposables.

### 3.2 Neuromonitoring Utilization and RLN Injury–Related Costs

**FIGURE 2:**
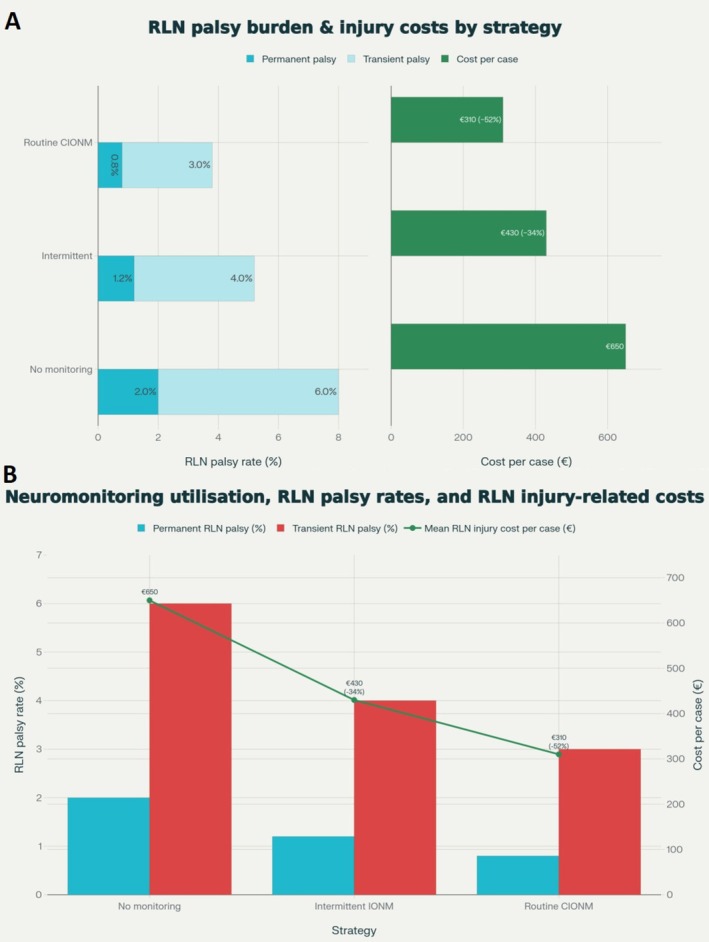
(A, B) RLN palsy burden, neuromonitoring utilization, and RLN injury‐related costs. (A) Panel A shows, for three intraoperative strategies (no neuromonitoring, intermittent IONM, routine CIONM), the rates of permanent and transient recurrent laryngeal nerve (RLN) palsy and the corresponding mean RLN injury management cost per thyroidectomy. The horizontal stacked bars on the left display permanent RLN palsy (dark cyan) and transient RLN palsy (light cyan) as percentages of all procedures, illustrating the progressive reduction in total RLN palsy burden from no monitoring (2.0% permanent, 6.0% transient) to intermittent IONM (1.2% permanent, 4.0% transient) and routine CIONM (0.8% permanent, 3.0% transient). The horizontal green bars on the right show the mean cost per case of RLN injury management (including rehabilitation, reinterventions, medicolegal expenses, and productivity losses), decreasing from €650 with no monitoring to €430 (−34%) with intermittent IONM and €310 (−52%) with routine CIONM. (B) Panel B presents the same three strategies on the x‐axis, plotting permanent RLN palsy rate (cyan bars) and transient RLN palsy rate (red bars) on the primary y‐axis, together with a green line on the secondary y‐axis indicating the mean RLN injury management cost per case. The figure highlights the association between increasing neuromonitoring utilization (from no monitoring to intermittent IONM to routine CIONM), progressively lower permanent and transient RLN palsy rates, and a stepwise reduction in RLN‐related costs per procedure (from €650 to €430 to €310), summarizing the clinical and economic benefits of routine CIONM. [Color figure can be viewed in the online issue, which is available at www.laryngoscope.com]

Higher neuromonitoring adoption markedly reduced RLN injury rates and related costs (Figure 2A,B). Visual identification alone resulted in 2.0% permanent and 6.0% transient palsy, with mean RLN management costs of €650 per case. Intermittent IONM (80% use) lowered rates to 1.2%/4.0% and costs to €430 (−34%), while routine CIONM further reduced palsy to 0.8%/3.0% and costs to €310 (−52% vs. no monitoring; −28% vs. intermittent). Across 6000 procedures, intermittent IONM prevented ~48 permanent and 120 transient injuries (~€1.3 M saved), and CIONM prevented an additional ~18 permanent and 60 transient injuries (~€0.7 M saved), reducing total RLN‐related expenditure from €3.9 to €1.9 M despite higher upfront monitoring costs.

### 3.3 Net Economic Impact and Comparative Performance of Monitoring Strategies

**FIGURE 3:**
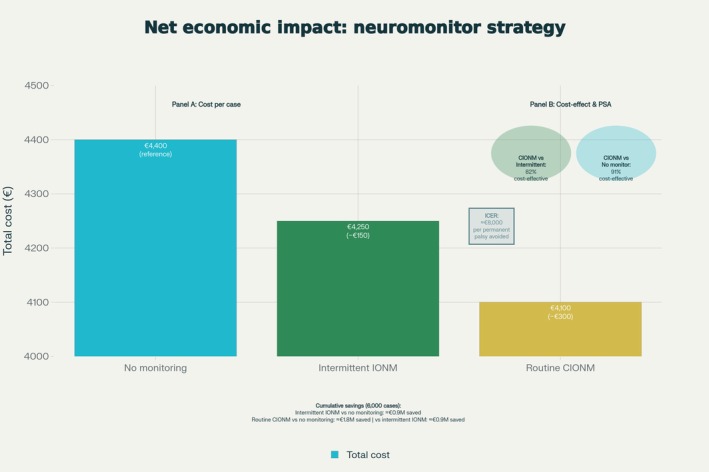
Net economic impact of neuromonitoring strategies. The mean total cost per thyroidectomy case (procedure plus RLN injury management) decreases from €4400 with no monitoring to €4250 with intermittent IONM and €4100 with routine CIONM, yielding cumulative savings of up to €1.8 million over 6000 procedures. Probabilistic analyses indicate that CIONM is cost‐effective compared to intermittent IONM in 82% of simulations and compared to no monitoring in 91% of simulations. [Color figure can be viewed in the online issue, which is available at www.laryngoscope.com]

When monitoring and RLN injury–related costs were combined, increased neuromonitoring utilization remained economically favorable (Figure 3). Visual identification alone generated a mean total cost of €4400 per case, compared with €4250 for intermittent IONM (net saving €150) and €4100 for routine CIONM (saving €300 vs. no monitoring and €150 vs. intermittent). Across 6000 cases, this corresponded to ~€1.8 million saved versus visual identification and ~€0.9 million versus intermittent IONM. CIONM consistently showed the best economic performance, with an ICER of ~€8000 per permanent RLN palsy avoided—well within accepted willingness‐to‐pay thresholds. Probabilistic analyses confirmed robustness: CIONM was cost‐saving or cost‐effective in 82% of simulations versus intermittent IONM and dominated visual identification in 91% of runs, indicating substantial reductions in both per‐case and overall RLN‐related economic burden.

### 3.4 Laryngoscopy

**FIGURE 4:**
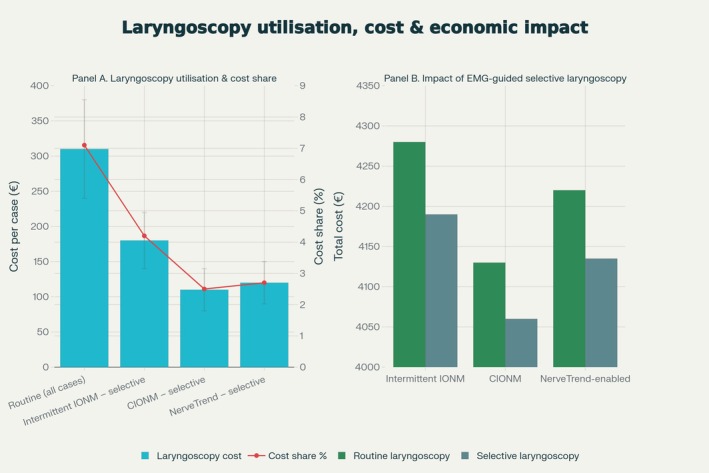
(A, B) Laryngoscopy utilization, cost share, and economic impact. Panel A shows the mean laryngoscopy cost per thyroidectomy case (bars, left y‐axis) and the proportion of total pathway costs attributable to laryngoscopy (line, right y‐axis) under different policies and neuromonitoring strategies. Routine pre‐ and postoperative laryngoscopy in all cases results in the highest per‐case expenditure (~€310; ~7.1% of total costs), whereas EMG‐guided selective policies progressively reduce both absolute laryngoscopy costs and their budget impact for intermittent IONM, CIONM, and NerveTrend‐enabled monitoring. Panel B illustrates the effect of switching from routine to EMG‐triggered selective laryngoscopy on overall mean total cost per case for each neuromonitoring modality. For intermittent IONM, CIONM, and NerveTrend, selective laryngoscopy lowers total costs by approximately €70–90 per procedure, leading to modest but consistent improvements in the cost‐effectiveness profile of neuromonitoring without altering the relative ranking of the strategies. [Color figure can be viewed in the online issue, which is available at www.laryngoscope.com]

In the reference scenario, routine pre‐ and postoperative laryngoscopy is performed in 100% of cases, representing 7.1% of total pathway costs (€310 per case; Figure 4A,B). Selective postoperative laryngoscopy triggered by intraoperative EMG deterioration markedly reduced utilization and costs: intermittent IONM required laryngoscopy in 42% of cases (€180), CIONM in 24% (€110), and NerveTrend in 27% (€120), lowering laryngoscopy‐related expenditure by 35%–65% across modalities (Figure 4A,B).

Incorporating laryngoscopy into the full economic model showed modest but consistent improvements in cost‐effectiveness. Switching from routine to selective laryngoscopy reduced total costs per case from €4280 to €4190 for intermittent IONM and from €4130 to €4060 for CIONM, improving ICERs by ~8%–10%. NerveTrend demonstrated similar reductions (€70–€90 per case) while maintaining a favorable cost–utility profile.

These findings align with contemporary guidelines advocating selective laryngeal examination targeted to high‐risk patients or abnormal intraoperative EMG findings, offering an efficient, value‐based approach that preserves safety while reducing unnecessary resource use.

### 3.5 Technical Failure of Neuromonitoring

#### 3.5.1 Direct Costs

**FIGURE 5:**
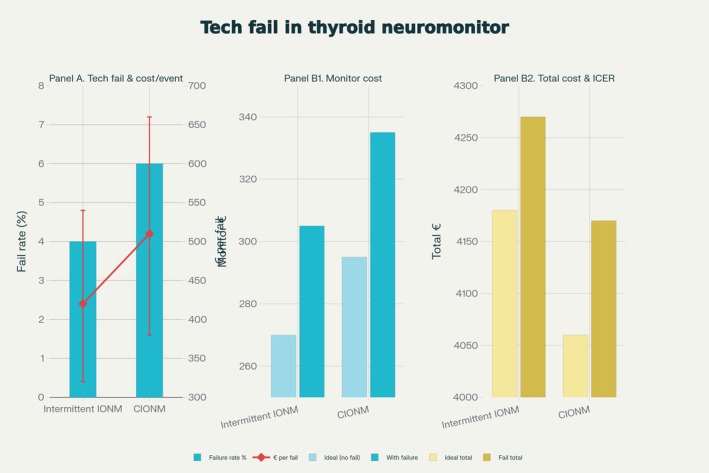
Technical failure in thyroid neuromonitoring. Panel A shows the simulated technical failure rates of intermittent IONM (4%) and CIONM (6%) (bars, left axis), and the corresponding incremental cost per failure event (red markers, right axis), with higher costs for CIONM reflecting more expensive consumables and longer troubleshooting. Panel B1 shows the increase in mean monitoring cost per case when technical failures are included (from €270 to €305 for intermittent IONM and from €295 to €335 for CIONM), while Panel B2 shows the resulting rise in total episode cost per case (from €4180 to €4270 and from €4060 to €4170, respectively) and the associated worsening of cost‐effectiveness, which partially offsets the economic benefits of neuromonitoring. [Color figure can be viewed in the online issue, which is available at www.laryngoscope.com]

Each technical failure added direct costs from prolonged anesthesia, extra staff time, additional disposables, and occasional repeat laryngoscopy. The mean incremental cost per failure was €420 (95% UI €320–€540) for intermittent IONM and €510 (95% UI €380–€660) for CIONM, reflecting higher CIONM consumable and troubleshooting requirements (Figure 5).

#### 3.5.2 Impact on Per‐Case and Pathway Costs

Across 6000 procedures, technical failure increased mean monitoring costs from €270 to €305 for intermittent IONM and from €295 to €335 for CIONM (13%–14%). Integrated into the full pathway, total episode costs rose by €90 per case for intermittent IONM (from €4180 to €4270) and by €110 for CIONM (from €4060 to €4170), offsetting 25%–35% of the savings otherwise achieved (Figure 5).

#### 3.5.3 Effect on Cost‐Effectiveness Metrics

Failure events worsened ICERs: intermittent IONM vs. visual identification rose from ~€8400 to ~€9700 per permanent palsy avoided, and CIONM vs. intermittent increased from ~€7500 to ~€8600. Although still within accepted willingness‐to‐pay thresholds, high failure rates (≥ 8%–10%) shifted many simulations into ranges where intermittent IONM was no longer cost‐effective and CIONM lost dominance in low‐volume settings.

### 3.6 Scenario Analyses and Mitigation Strategies

Failure frequency showed a near‐linear cost effect, with each 1% increase raising monitoring expenditure by ~€8–€10 per case. Incorporating structured training, standardized troubleshooting, and reliable stock management halved simulated failure rates, restoring much of the lost cost‐effectiveness and confirming technical reliability as a major determinant of neuromonitoring value.

### 3.7 Manufacturer‐Focused Results

#### 3.7.1 Manufacturer‐Stratified Monitoring Costs

Across Medtronic, Inomed, Dr. Langer Medical, NCC Medical, Natus, and Neurovision, mean monitoring costs were similar when matched by modality and electrode type. With ≥ 80% utilization and ≥ 300 annual cases, per‐case expenditure ranged €250–€310 without significant between‐platform differences (Table 1).

**TABLE 1:** Manufacturer‐stratified mean monitoring costs per case.

Manufacturer	Monitoring modality/configuration	Mean monitoring cost per case (€)^a^	Utilization level^b^	Centre annual volume^c^	Between‐platform differences^d^
Medtronic	Base‐case IONM configuration	260–300	High (≥ 80% thyroidectomies)	≥ 300 cases/year	Not significant
Inomed	Base‐case IONM configuration	250–285	High (≥ 80% thyroidectomies)	≥ 300 cases/year	Not significant
Dr. Langer Medical	Base‐case IONM configuration	260–290	High (≥ 80% thyroidectomies)	≥ 300 cases/year	Not significant
NCC Medical	Base‐case IONM configuration	250–310	High (≥ 80% thyroidectomies)	≥ 300 cases/year	Not significant
Natus	Base‐case IONM configuration	230–280	High (≥ 80% thyroidectomies)	≥ 300 cases/year	Not significant
Neurovision	Base‐case IONM configuration	250–285	High (≥ 80% thyroidectomies)	≥ 300 cases/year	Not significant

^a^ Mean per‐case direct monitoring costs, expressed in € for the model reference year.

^b^ High utilization scenario: ≥ 80% of thyroidectomies monitored with IONM.

^c^ Centre‐level surgical volume scenario: ≥ 300 thyroidectomy cases per year.

^d^ No statistically significant between‐platform differences after adjustment for surgical volume and case‐mix.

#### 3.7.2 Electrode Configuration and Per‐Case Costs

Integrated EMG endotracheal tubes generated the highest disposable costs (€380–€420 per case), whereas stitched, surface, or needle‐based configurations reduced electrode costs to €60–€120 and total monitoring expenditure to €230–€270 at high utilization, with no loss of EMG reliability (Table 2).

**TABLE 2:** Electrode configuration costs.

Electrode configuration	Electrode cost per case (€)^a^	Total monitoring cost per case at high utilization (€)^b^	EMG recording reliability^c^
Integrated EMG endotracheal tube	380–420	380–420	Maintained (reference configuration)
Stitched surface EMG electrodes	60–120	230–270	Maintained
Adhesive surface EMG electrodes	60–120	230–270	Maintained
Percutaneous needle electrodes (cricothyroid region)	60–120	230–270	Maintained
Transcartilaginous needle electrodes (cricothyroid region)	60–120	230–270	Maintained

^a^ Mean per‐case electrode expenditure, consistent with published unit prices for EMG tubes and alternative electrodes.

^b^ High‐utilization scenario (≥ 80% of thyroidectomies monitored), centre volume ≥ 300 cases/year; includes all monitoring‐related disposables and capital allocation where applicable.

^c^ No relevant loss of EMG signal reliability observed across non‐tube configurations in the simulations.

#### 3.7.3 Intermittent IONM Versus CIONM

Across manufacturers, intermittent IONM cost ~€260 per case in high‐volume centres versus ~€290 for CIONM. However, incorporating RLN‐related costs reduced total per‐procedure expenditure to €4050 with CIONM compared with €4180 for intermittent IONM, yielding ICERs of €7000–€9000 per permanent palsy avoided (Table 3).

**TABLE 3:** Intermittent IONM Versus CIONM cost and ICER results.

Strategy	Monitoring cost per case (€)	Total cost per thyroidectomy^a^ (€)	Incremental cost vs intermittent (€)	Incremental effectiveness^b^ (permanent RLN palsies averted)	ICER (€/permanent palsy averted)
Intermittent IONM	260	4180	—	—	—
Continuous IONM (CIONM)	290	4050	−130	0.015–0.020	7000–9000

^a^ Includes monitoring costs plus RLN palsy–related expenditures per thyroidectomy.

^b^ Incremental effectiveness expressed as permanent RLN palsies averted per thyroidectomy when moving from intermittent IONM to CIONM; range consistent with ICER of €7000–€9000 per permanent palsy averted given the reported cost difference.

#### 3.7.4 Impact of Utilization and Volume

At low utilization (20%), mean monitoring costs exceeded €450 per case across platforms. Routine use (≥ 80%) in high‐volume centres lowered monitoring costs by ~35%–40% to €260–€300 and amplified downstream savings from avoided RLN palsy, especially for CIONM and NerveTrend‐assisted strategies (Table S2).

#### 3.7.5 Manufacturer‐Specific Cost‐Effectiveness

In high‐volume endocrine units, Medtronic APS‐enabled CIONM/NerveTrend, Inomed C2/C2 Xplore, and Langer AVALANCHE‐based CIONM all demonstrated superior cost‐utility versus intermittent IONM, with slightly higher monitoring costs (€290–€310) offset by the greatest reductions in permanent RLN palsy and consistently positive incremental net benefit across willingness‐to‐pay thresholds.

#### 3.7.6 Trend‐Monitoring Platforms

Automated nerve trend‐monitoring (e.g., Medtronic NerveTrend) incurred intermediate costs (€280–€300), with RLN injury rates and cost‐effectiveness closely approximating full CIONM. These platforms had a high probability of being cost‐effective relative to intermittent IONM, particularly where adoption of dedicated CIONM hardware is limited (Table S3).

## 4 Discussion

This analysis demonstrates that IONM reduces per‐case monitoring expenditure and substantially mitigates the downstream economic burden of RLN injury. Rather than a binary “use vs no‐use” debate, these findings support comparing monitoring modalities, utilization strategies, and platform ecosystems based on value delivered [37].

### 4.1 CIONM as a Core Patient‐Safety Technology

CIONM should be regarded as a patient‐safety technology, not an optional adjunct. By preventing additional transient and permanent RLN palsies beyond intermittent IONM, CIONM yields lower total episode costs despite higher upfront hardware and disposable expenses, with ICERs well below accepted willingness‐to‐pay thresholds. High‐volume centres are best positioned to realize these benefits. Where dedicated hardware is unavailable, advanced intermittent and trend‐aware workflows can approximate the economic value of CIONM.

### 4.2 Comprehensive Modeling and Methodological Strengths

A key strength of this study is the unified cost–utility model comparing all major neuromonitoring modalities and platforms used in thyroid surgery. Integrating full‐pathway micro‐costing with device‐specific parameters and QALY outcomes provides a nuanced assessment of trade‐offs among capital investment, disposables, staff time, and avoided nerve‐injury costs. Manufacturer‐level, electrode‐configuration, and laryngoscopy‐strategy analyses increase the practical relevance of findings for procurement, workflow optimisation, and reimbursement discussions.

### 4.3 Platform Cost Convergence and Value‐Based Procurement

Once modality, configuration, and utilization volume were standardized, per‐case monitoring costs were broadly similar across manufacturers. With market maturation narrowing price differences, economic decisions should prioritize system reliability, workflow integration, software capabilities, and maintenance support rather than nominal acquisition cost.

### 4.4 Utilization, Technical Reliability, and the Hidden Cost of Failure

Utilization intensity and system reliability were major determinants of economic performance. Routine monitoring in high‐volume environments reduced costs by over one third relative to selective low‐volume use. Importantly, even modest technical failure rates substantially eroded the economic benefit of IONM and CIONM. Ensuring uninterrupted signal integrity requires coordinated work among surgeons, anesthetists, and technical staff, supported by structured training and robust maintenance protocols [9, 38]. Technical reliability should be considered a core determinant of programme efficacy.

### 4.5 Selective Laryngoscopy and Economic Optimisation

Routine pre‐ and postoperative laryngoscopy remains important [39], but EMG‐guided selective postoperative laryngoscopy can further improve cost‐effectiveness without compromising safety. When both RLNs show stable, high‐amplitude signals at the end of bilateral thyroidectomy, omitting routine postoperative laryngoscopy may represent a rational, value‐aligning strategy [40].

### 4.6 Limitations and Future Directions

This evaluation is based on a decision analytic model that necessarily relies on assumptions regarding RLN palsy rates, relative risk reductions achieved with IONM and CIONM, technical failure frequencies, laryngoscopy policies, and cost inputs, some of which are informed by heterogeneous literature and context‐specific institutional data. Although extensive deterministic and probabilistic sensitivity analyses were conducted across wide parameter ranges—including low permanent palsy rates and very low technical failure frequencies typical of high‐volume endocrine units—and consistently confirmed the qualitative robustness of the main conclusions, the results should still be interpreted as scenario‐based estimates rather than direct observational findings. In particular, the chosen base case permanent palsy probabilities and technical failure rates are deliberately conservative and may overestimate the incremental benefit of neuromonitoring in centers with ultra‐low baseline complication rates and highly optimized workflows. Local calibration of the model using center‐specific RLN outcomes, failure statistics, and cost schedules is therefore recommended when using these data to support procurement, implementation, or reimbursement decisions.

A strength of the proposed framework is that all key parameters are fully transparent and re‐parameterizable, allowing institutions to substitute their own volumes, costs, RLN rates, and technical performance metrics to generate tailored cost–utility estimates.

## 5 Conclusions

Intraoperative neuromonitoring—especially CIONM and advanced trend‐based systems—offers clear clinical and economic advantages in thyroid surgery when implemented in protocol‐driven, high‐volume units. These benefits depend on technical reliability and coordinated teamwork to maintain stable signals. Selective EMG‐guided postoperative laryngoscopy further enhances cost‐effectiveness without compromising safety.

## Funding

The authors have nothing to report.

## Conflicts of Interest

The authors declare no conflicts of interest.

## Supporting information


**Table S1:** Intraoperative neuromonitoring platforms for thyroid surgery: manufacturer specifications and technical features.
**Table S2:** Estimated saving from avoided RLN palsy per case (€): mean saving per thyroidectomy due to reduced permanent RLN palsy, including hospital, rehabilitation, phonosurgery, medico‐legal, and productivity loss costs.
**Table S3:** Trend‐monitoring platforms—simulated costs and outcomes.

**Data S1:** Patient pathway analysis in thyroidectomy with intraoperative neural monitoring. Comprehensive methodological framework for cost‐utility analysis.

**Data S2:** Spreadsheet providing an overview of costs.

**Data S3:** Study arms and analyses.

## Data Availability

The data that support the findings of this study are available on request from the corresponding author. The data are not publicly available due to privacy or ethical restrictions.
