# Supplementary material for: Value‐Based Neuromonitoring in Thyroidectomy: A Comprehensive Cost–Utility Analysis
Source: Laryngoscope. 2026 Mar 31;136(7):3271–83. doi: 10.1002/lary.70536 (PMC13253177; doi:10.1002/lary.70536)
Supplement: Supplementary file 1 — Table S1: Intraoperative neuromonitoring platforms for thyroid surgery: manufacturer specifications and technical features. Table S2: Estimated saving from avoided RLN palsy per case (€): mean saving per thyroidectomy due to reduced permanent RLN palsy, including hospital, rehabilitation, phonosurgery, medico‐legal, and productivity loss costs. Table S3: Trend‐monitoring platforms—simulated costs and outcomes. [file LARY-136-3271-s002.docx]

## ****Supplementary Table S1.** Intraoperative Neuromonitoring Platforms for Thyroid Surgery: Manufacturer Specifications and Technical Features**

| **Manufacturer** | **Country** | **Primary Platform(s)** | **Monitoring Modality** | **Recording Configuration** | **Key Technical Features** | **Advanced Functions** | **Thyroid-Specific Accessories** |
| --- | --- | --- | --- | --- | --- | --- | --- |
| **Medtronic** | USA | NIM-Response 3.0, NIM-Neuro 3.0, NIM Vital | Intermittent + Continuous (CIONM) | EMG endotracheal tubes (TriVantage, APS-enabled), APS vagus electrodes, surface electrodes | 4-channel simultaneous monitoring; real-time visual and auditory feedback; color-coded status display (green/yellow/red) | NerveTrend® automated EMG trend analysis; proprietary nerve condition monitoring algorithm; wireless data transmission | TriVantage EMG tubes (multiple sizes 6–10 mm); APS vagus electrodes; Nervassure electrodes |
| **Inomed Medizintechnik** | Germany | C2, C2 Xplore | Intermittent + Continuous (CIONM) | Integrated EMG endotracheal tubes (ALM Tube), vagal electrodes, standardized stimulation probes | Multichannel EMG recording; ALM (Autologous Laryngeal Monitoring) technology with intelligent Channel Select Software; integrated database for automatic data storage | Smart trend software for automated signal analysis; 2D LED scanner for rapid patient data retrieval; digital integration with hospital information systems; wireless connectivity | ALM Tube (universal compatibility); dedicated thyroid/ENT modules; multichannel EMG recording accessories |
| **Dr. Langer Medical** | Germany | AVALANCHE SI 2 | Intermittent IONM | Needle and surface electrodes; cricothyroid muscle electrodes; procedure-specific probes | Up to 8 measurement channels; modular architecture; touchscreen interface with hygiene-optimized glass front (Windows 10 IoT); enhanced documentation with trend reporting | Multimodal neuromonitoring capability; expanded connectivity (LAN/WLAN); trend analysis reporting; compatible with hospital information systems | Needle electrodes for cricothyroid insertion; surface electrodes for laryngeal application; endocrine neck surgery-specific stimulation and recording configurations |
| **NCC Medical** | China | NCC Smart IONM, Cynapse IONM | Intermittent + Continuous (configurable) | Configurable channels for RLN, cranial, spinal monitoring; EMG electrodes; integrated consumables | 8 recording + 2 stimulation channels (Smart IONM); 16/32 channels available (Cynapse); real-time impedance testing; event-, stimulus-, and EMG-based audio feedback | Default presets based on surgical type; dual-screen display capability; synchronized microscope/video feed; one-wire connection reducing setup errors; anti-interference integrated consumables | RLN-specific electrode configurations; thyroidectomy-optimized templates; customizable parameters for local practice variations |
| **Natus** | USA | Generic multipurpose intraoperative neuromonitoring systems | Intermittent + Continuous (system-dependent) | Integrates with universal electrode systems; compatible with multiple electrode configurations | Variable specifications depending on specific platform model and configuration; broad compatibility with standard electrodes | Platform-dependent; generally supports standard EMG recording and analysis; integration with third-party electrodes | Compatible with universal laryngeal electrodes; configurable per institutional protocol; supports multiple electrode types |
| **Neurovision Medical Products** | USA | Dragonfly Laryngeal Electrode, Universal EMG electrodes | Intermittent monitoring (electrode-based interface) | Adhesive surface laryngeal electrodes (Dragonfly, Lantern design); universal compatibility with multiple consoles | Integrates with any IONM console; patented surface electrode design; fits endotracheal tubes 2.5–10 mm ID; 20-year clinical validation; low-impedance contact; depth markings for optimal positioning | Universal cross-platform standardization; enables interoperability with Medtronic, Inomed, Natus, and other systems | Dragonfly 1-channel laryngeal electrode (multiple ETT sizes); universal EMG electrodes; pediatric and adult sizes; proven dual-channel configurations |

**Supplementary Table S2.** Estimated saving from avoided RLN palsy per case (€): Mean saving per thyroidectomy due to reduced permanent RLN palsy, including hospital, rehabilitation, phonosurgery, medico-legal, and productivity loss costs.

| **Scenario** | **Proportion of thyroidectomies with IONM/CIONM** | **Centre annual volume (thyroidectomies)** | **Mean monitoring cost per case (€)** | **Relative change vs high-utilisation (%)** | **Estimated saving from avoided RLN palsy per case (€)*** | **Notes on downstream RLN palsy–related costs†** |
| --- | --- | --- | --- | --- | --- | --- |
| Low utilisation, mixed volume centres | 20% | <300–300 | >450 | Reference | 0–50 (IONM/CIONM, any technology) | Limited effect on RLN palsy burden; capital poorly amortised. |
| High utilisation, high-volume centres – Intermittent IONM | ≥80% | ≥300 | 260–280 | −35% to −40% | ~80–100 | Moderate reduction in permanent RLN palsy and related expenditures. |
| High utilisation, high-volume centres – CIONM | ≥80% | ≥300 | 280–300 | −35% to −40% | ~120–150 | Greater reduction in permanent RLN palsy, lowering total cost per case. |
| High utilisation, high-volume centres – CIONM + NerveTrend | ≥80% | ≥300 | 280–300 | −35% to −40% | ~150–180 | Enhanced early-warning and decision support; maximised savings per case. |

*Mean saving per thyroidectomy from reduced permanent RLN palsy, incorporating hospital, rehabilitation, medico‑legal and productivity costs; values are indicative and technology‑specific within the model structure.
†Downstream savings increase with higher utilisation and centre volume, and are greatest for CIONM and NerveTrend‑assisted strategies due to larger relative reductions in permanent RLN injury rates.

**Supplementary Table S3.** Trend-monitoring platforms – simulated costs and outcomes

| **Strategy / platform** | **Monitoring cost per case (€)** | **Relative monitoring cost vs intermittent IONM** | **RLN injury rates vs CIONM*** | **Cost‑effectiveness vs intermittent IONM†** | **Context of use** |
| --- | --- | --- | --- | --- | --- |
| Conventional intermittent IONM | ~260 | Reference | Higher | Reference | Baseline strategy in high‑volume centres. |
| Full CIONM (dedicated continuous hardware) | ~290 | Higher (↑ capital and disposables) | Lower | Favourable ICER per permanent RLN palsy averted | Requires dedicated CIONM console and continuous vagal electrode. |
| Automated trend-monitoring (NerveTrend) | 280–300 | Intermediate (↑ software and disposables) | Comparable to CIONM in simulations | High probability of being cost‑effective vs intermittent IONM | Particularly attractive where CIONM hardware adoption is limited by budget or workflow constraints. |

* RLN injury rates for automated trend‑monitoring were comparable to those of full CIONM at cohort level.
†Modelled cost‑effectiveness indices (e.g. cost per permanent RLN palsy averted) for NerveTrend fell in a similar range to CIONM, with a high probability of cost‑effectiveness versus standard intermittent IONM in probabilistic analyses.
