# Supplementary material for: Value‐Based Neuromonitoring in Thyroidectomy: A Comprehensive Cost–Utility Analysis
Source: Laryngoscope. 2026 Mar 31;136(7):3271–83. doi: 10.1002/lary.70536 (PMC13253177; doi:10.1002/lary.70536)
Supplement: Supplementary file 2 — Data S1: Patient pathway analysis in thyroidectomy with intraoperative neural monitoring. Comprehensive methodological framework for cost‐utility analysis. [file LARY-136-3271-s003.docx]

**Supplementary Material 2:** Patient Pathway Analysis in Thyroidectomy with Intraoperative Neural Monitoring. Comprehensive Methodological Framework for Cost-Utility Analysis

**1. Detailed Patient Pathway Mapping and Process Decomposition**

**1.1 Comprehensive Pathway Architecture**

Patient pathway analysis in thyroidectomy with intraoperative neural monitoring (IONM/CIONM) encompasses a continuum of care extending from initial clinical presentation through long-term follow-up. This pathway is systematically decomposed into discrete, time-sequenced process steps, each linked to identifiable resource consumption and cost drivers.

**Preoperative Phase (Weeks -12 to -1):**

- Initial endocrine consultation: Clinical evaluation, comorbidity assessment, indication clarification
- Surgical consultation: Procedural explanation, consent documentation, risk stratification
- Anesthesiology assessment: Medical optimization, airway evaluation, ASA classification
- Laryngoscopy (baseline and repeat): Direct or flexible visualization of vocal cord function; documentation of preoperative voice quality
- Laboratory investigations: Complete blood count, comprehensive metabolic panel, coagulation studies, thyroid function tests (TSH, free T4), serum calcium, phosphate, parathyroid hormone (PTH), 25-hydroxy vitamin D
- Imaging studies: Neck ultrasonography (thyroid volume, nodule characterization, lymph node assessment); computed tomography or magnetic resonance imaging when central compartment involvement or retrosternal extension suspected
- Outpatient preoperative clinic: Final clearance, medication reconciliation, anesthesia optimization
- Staff time quantification: Surgeon time (30–45 minutes per patient), anesthesiologist time (20–30 minutes), nursing time (45–60 minutes), laboratory technician time, imaging technician time

**Intraoperative Phase (Day of Surgery, 0–4 hours):**

- Anesthetic induction and monitoring: Airway management, placement of endotracheal intubation with recording surface electrodes (for IONM), baseline hemodynamic monitoring
- Operative room setup: IONM equipment positioning, calibration, troubleshooting protocols
- Surgical procedure: Incision, flap creation, identification of anatomical landmarks, dissection of critical structures (recurrent laryngeal nerve, superior laryngeal nerve, parathyroid glands)
- Neural monitoring: Electrode placement confirmation, baseline signal acquisition, intermittent or continuous monitoring per protocol
- Thyroidectomy execution: Hemithyroidectomy (unilateral lobe resection ± isthmus) or total thyroidectomy (bilateral lobes ± isthmus)
- Hemostasis and closure: Wound inspection, drainage placement (if indicated), fascial and skin closure
- Operative time documentation: Total surgical time, IONM setup/troubleshooting time, blood loss quantification
- Staff time: Surgeon (90–150 minutes), anesthesiologist (90–150 minutes), anesthetic technician (entire operative duration), surgical nurses (entire operative duration), IONM technician (entire operative duration), biomedical engineer on-call (if equipment issues)
- Equipment and consumables: Reusable operating room instrumentation, electrosurgical unit consumables, IONM electrodes and probes (device-specific), recording cables, surgical drapes, irrigation solutions, sutures, hemostatic agents

**Immediate Postoperative Phase (Hours 0–24, Recovery and Ward Stay):**

- Recovery room monitoring: Emergence from anesthesia, hemodynamic stability, pain assessment, drain output monitoring
- Vocal function assessment: Bedside voice quality assessment, screening for stridor or dysphonia
- Calcium and PTH monitoring: Serum calcium and ionized calcium (immediate postoperative period and post-operative day 1), PTH levels, clinical assessment for hypocalcemia symptoms (paresthesia, tetany)
- Complications management: Hematoma evacuation (if required), cervical drain management, transfusion (if significant blood loss)
- Ward stay: High-dependency or standard ward admission; continuous monitoring of vital signs, drain output, wound integrity, pain control
- Analgesic and antiemetic therapy: Opioid analgesics (morphine, oxycodone equivalents), non-opioid analgesics (acetaminophen, NSAIDs), antiemetics (ondansetron, metoclopramide)
- Nutritional support: Diet advancement as tolerated; swallowing assessment if dysphagia suspected
- Staff time: Surgical nursing (8–16 hours), anesthesia recovery staff (2–4 hours), ward nursing (remainder of admission), surgical residents (assessments), attending surgeon (daily rounds)
- Bed occupancy and facilities: Operating room time, recovery room bed-days, ward bed-days, equipment maintenance and depreciation

**Early Postoperative Follow-up Phase (Weeks 1–6):**

- Postoperative clinic visit (1–2 weeks): Wound inspection, drain removal assessment, voice and swallowing function evaluation
- Repeat laryngoscopy (if indicated): Formal assessment of vocal cord mobility; documentation of transient versus permanent nerve injury
- Laboratory rechecks: Serum calcium, PTH, thyroid function (TSH, free T4)
- Speech therapy referral (if dysphonia or dysphagia present)
- Imaging (if complications suspected): Ultrasound or CT for hematoma, abscess, or anatomical assessment
- Endocrine follow-up (weeks 4–6): Initiation of thyroid hormone replacement (if total thyroidectomy), dose titration, monitoring of symptoms
- Staff time: Surgeon (20–30 minutes per clinic visit), nurses (15–20 minutes), laryngologists (30–45 minutes if laryngoscopy performed), speech pathologist (30–60 minutes if therapy needed), laboratory personnel
- Imaging technician time

**1.2 Resource Use Triangulation and Validation**

Resource utilization data were derived from three complementary, independent sources to ensure accuracy and completeness:

**Institutional Administrative Databases:**

- Hospital information system (HIS) and electronic health record (EHR) queries capturing length of stay, bed-days by ward type, operative time logs
- Pharmacy records documenting medication dispensations (type, quantity, cost)
- Laboratory information system capturing all tests ordered, performed, and reported
- Imaging department records of ultrasonography, CT, MRI procedures with technical specifications
- Finance and billing department data on equipment depreciation, facility overhead allocation

**Theatre Information and Anesthesia Records:**

- Detailed operative logs documenting start/stop times, personnel present, specific procedures performed
- Anesthesia records capturing induction time, maintenance, emergence, medications administered
- IONM device logs recording setup time, signal quality, troubleshooting events, duration of monitoring
- Consumable tracking from theatre supply systems (electrode batches, device-specific tubes, etc.)

**Structured Stakeholder Interviews:**
Systematic interviews with 15–20 key personnel across surgical, anesthetic, nursing, and biomedical engineering teams:

- Surgeons (n=3): Operative time estimates, device preference, learning curve considerations
- Anesthesiologists (n=2): Setup time, monitoring requirements, equipment issues frequency
- Surgical nurses (n=3): Setup time, intraoperative support, complication management protocols
- IONM technicians (n=2): Equipment setup/calibration time, troubleshooting frequency, training requirements
- Biomedical engineers (n=2): Equipment maintenance schedules, failure rates, repair timelines
- Laboratory staff (n=2): Test ordering patterns, sample processing time
- Ward nursing (n=2): Postoperative monitoring intensity, complication prevalence

Interview guides employed open-ended questions with standardized worksheets to quantify:

- Time allocations (in minutes) for specific tasks
- Frequency of procedural variations or complication responses
- Equipment utilization patterns
- Subjective assessment of workload and resource adequacy

**2. Cost Driver Specification and Quantification**

**2.1 Personnel Costs**

Personnel costs represent the largest direct component of thyroidectomy pathway costs. Costs are calculated using standardized labor rates (derived from institutional human resources databases and national wage statistics) multiplied by task-specific time allocations:

**Surgical personnel:**

- Consultant surgeon: Operative time (90–150 min), preoperative consultation (30–45 min), postoperative rounds and clinic follow-up (30–60 min over 6-week period)
- Surgical resident/fellow: Operative time (90–150 min), preoperative assessment (15–30 min), postoperative ward rounds (20–40 min), early clinic visit (15–20 min)
- Scrub nurse: Setup (15–30 min), operative time (90–150 min), room turnover (10–15 min)
- Circulating nurse: Setup and patient preparation (30–45 min), operative time (90–150 min), room turnover (10–15 min)

**Anesthetic personnel:**

- Anesthesiologist: Preoperative evaluation (20–30 min), operative room time (90–150 min)
- Anesthetic technician: Setup (15–20 min), operative time (90–150 min)
- Recovery staff: Recovery room time (30–60 min)

**Intraoperative monitoring personnel:**

- IONM technician: Setup and calibration (15–30 min), operative time (90–150 min), troubleshooting events (0–30 min per case)
- Biomedical engineer (on-call for major equipment issues): Time per case (0–60 min, dependent on device-related complications)

**Postoperative ward and clinic staff:**

- Ward nurses: Per diem allocation for bed monitoring, medications, drain management (2–4 hours per patient admission)
- Clinic nurses: Scheduling, vital signs, wound assessment (15–20 min per clinic visit)
- Laryngologists: Laryngoscopy performance if early dysphonia assessment needed (30–45 min)
- Speech pathologists: Consultation and therapy if dysphagia/dysphonia present (30–60 min)

**Laboratory and imaging personnel:**

- Laboratory technicians: Sample collection, processing, quality control (15–20 min per patient pathway)
- Imaging technicians: Ultrasonography or CT (20–40 min per procedure, equipment time)

**2.2 Consumable and Disposable Costs**

**Intraoperative neural monitoring consumables (device-specific):**

Medtronic NIM Response™ System:

- Reusable electrodes: £500–800 per set; typical lifespan 50–100 procedures
- Disposable surface electrodes: £15–25 per set per case
- Recording cables and connectors: £300–500 per set; lifespan 100+ procedures
- Software licenses: £5,000–8,000 annually per operating theatre

Inomed ISIS IOM™ System:

- Needle electrodes: £8–12 per pair per case (inserted into cricothyroid muscle)
- Reusable stimulus probes: £400–600 per set; lifespan 50+ cases
- Disposable cables: £10–18 per case
- Software maintenance: £4,000–6,500 annually

Natus Neuro-Eclipse™:

- Disposable EMG electrodes: £12–20 per set per case
- Reusable nerve stimulation probes: £350–550 per set
- Recording modules: £200–400 per set (depreciation amortized)
- Annual support contract: £3,500–5,500

**Surgical consumables:**

- Electrosurgical generator: £20–35 per case (depreciation)
- Hemostatic agents (FloSeal®, Surgicel®): £30–60 per case
- Sutures (for closure): £15–25 per case
- Surgical drapes and instruments: £50–80 per case
- Irrigation solutions (normal saline, epinephrine): £5–10 per case

**Anesthetic consumables:**

- Endotracheal tubes with recording electrodes: £20–35 per case
- Anesthetic drugs (induction, maintenance, reversal): £40–70 per case
- Monitoring cables and disposable probes: £10–15 per case

**Postoperative consumables:**

- Medications (analgesics, antiemetics): £15–30 per patient
- Wound dressings and drain management supplies: £20–35 per patient
- Laboratory reagents and materials: £5–10 per patient per test episode

**2.3 Equipment Costs (Depreciation and Maintenance)**

**Capital equipment costs amortized over expected lifespan:**

Intraoperative Neural Monitoring Systems (comparison of major manufacturers):

| **System** | **Capital Cost (€)** | **Lifespan (years)** | **Annual Depreciation (€)** | **Annual Maintenance (€)** | **Total Annual Cost (€)** |
| --- | --- | --- | --- | --- | --- |
| Medtronic NIM Response | 120,000–150,000 | 7–10 | 15,000–18,000 | 5,000–8,000 | 20,000–26,000 |
| Inomed ISIS IOM | 100,000–130,000 | 7–10 | 12,500–16,000 | 4,500–7,000 | 17,000–23,000 |
| Langer Neuro-Monitor | 80,000–110,000 | 7–10 | 10,000–14,000 | 3,500–6,000 | 13,500–20,000 |
| NCC Medical M1Pro | 90,000–120,000 | 7–10 | 11,000–15,000 | 4,000–6,500 | 15,000–21,500 |
| Natus Neuro-Eclipse | 110,000–140,000 | 7–10 | 13,500–17,000 | 5,000–7,500 | 18,500–24,500 |

**Other operative room equipment (shared allocation per thyroidectomy case):**

- Operating table and positioning accessories: €5–10 per case (depreciation)
- Anesthesia workstation: €8–12 per case
- Surgical lighting and ceiling-mounted equipment: €3–5 per case
- Room ventilation and climate control: €2–4 per case
- Facility maintenance and overhead (rent, utilities, insurance): €15–25 per case

**Facility and overhead costs:**

- Operating theatre rent/lease (per hour): €100–200 per hour
- Hospital administration, management, human resources: Allocation €5–10 per case
- Sterilization and instrument reprocessing: €20–30 per case

**3. Comparative Pathway Analysis by Monitoring Strategy**

**3.1 Visual Identification Alone (No IONM)**

**Pathway specifications:**

- No preoperative IONM training or accreditation required
- No intraoperative equipment setup or troubleshooting
- Minimal additional consumables (standard surgical supplies only)
- Relies on anatomical landmarks and surgeon experience
- Higher risk of undetected nerve injury (particularly in redo surgery or central compartment disease)

**Cost profile (per case):**

- Personnel: Surgeon (120 min), anesthesiologist (120 min), nursing staff (180 min) = €800–1,200 in labor
- Consumables (non-IONM): €150–200
- Equipment depreciation: €40–60
- **Total direct costs: €1,100–1,450 per case**

**Postoperative cost implications:**

- Higher rates of RLN injury (1.5–2.5% in high-volume centres)
- Increased need for laryngoscopic assessment (€200–400 per patient if performed)
- Speech therapy referrals in 8–15% of cases (€500–1,500 per course)
- Medicolegal costs (approximately €50,000–150,000 per claim on rare occasions)

**3.2 Intermittent IONM**

**Pathway specifications:**

- Endotracheal tube with integrated recording electrodes
- Stimulus probe placed on RLN or vagus nerve during dissection
- Intermittent test stimulation (every 15–30 minutes or at key decision points)
- Requires IONM technician presence and interpretation
- Moderate training requirements for surgical team

**Cost profile (per case):**

- Personnel: Surgeon (120 min), anesthesiologist (120 min), IONM technician (130 min), nursing staff (180 min) = €1,200–1,600 in labor
- IONM consumables: €40–70 per case
- Equipment depreciation: €60–100 per case (allocated based on annual case volume)
- **Total direct costs: €1,450–1,900 per case**

**Operational considerations:**

- Setup time: 15–25 minutes (added to operative time)
- Troubleshooting events: 5–10% of cases require probe repositioning (adds 10–20 min per event)
- Signal quality dependent on electrode placement and muscle paralysis reversal

**3.3 Continuous Intraoperative Neural Monitoring (CIONM)**

**Pathway specifications:**

- Endotracheal tube with integrated recording electrodes and vagal needle electrodes
- Continuous nerve stimulus (usually 1–2 mA, 4–5 Hz) throughout procedure
- Real-time EMG amplitude and latency trending
- Alerts for loss of signal (LOS: amplitude <50 µV) or conduction impairment (≥50% amplitude decrease with ≥10% latency increase)
- Requires experienced IONM technician and surgeon training
- Higher capital equipment costs; most comprehensive monitoring modality

**Cost profile (per case):**

- Personnel: Surgeon (120 min), anesthesiologist (120 min), IONM technician (150 min), nursing staff (180 min) = €1,400–1,800 in labor
- CIONM consumables: €60–95 per case (more electrodes, continuous recording media)
- Equipment depreciation: €120–180 per case (highest-cost systems)
- Software and license allocation: €10–15 per case
- **Total direct costs: €1,750–2,300 per case**

**Operational considerations:**

- Setup time: 25–35 minutes
- Troubleshooting events: 8–15% of cases (electrode repositioning, baseline reestablishment)
- Real-time trending capability may reduce undetected injuries
- Increased educational burden for OR staff

**3.4 Pathway-Specific Cost Drivers: Complication Management**

**Recurrent laryngeal nerve injury management:**

Transient RLN palsy (anticipated recovery within 6 months):

- Laryngoscopy for confirmation (€200–350 per procedure)
- Voice therapy (typically 8–12 sessions): €1,200–1,800
- Voice handicap index and quality-of-life assessments: €50–100
- Subtotal: €1,500–2,250 per case

Permanent RLN injury requiring intervention:

- Voice therapy (extended course, 20+ sessions): €2,000–3,000
- Vocal cord injection (calcium hydroxylapatite or other agents): €2,500–4,000
- Laryngeal framework surgery (medialization thyroplasty) if pursued: €8,000–15,000
- Subtotal: €3,000–20,000+ depending on intervention strategy

**Hypoparathyroidism (hypocalcemia):**

Transient hypoparathyroidism (resolution within 6 months):

- Emergency laboratory testing (calcium, PTH, magnesium): €50–100
- Intravenous calcium gluconate (if symptomatic acute hypocalcemia): €100–150
- Oral calcium and calcitriol supplementation: €50–100 per month
- Follow-up laboratory monitoring (calcium, PTH): €100–150 over 6-month period
- Subtotal: €300–500 per case

Permanent hypoparathyroidism (ongoing supplementation required):

- Long-term oral calcium and calcitriol supplementation: €80–150 per month
- Quarterly laboratory monitoring: €200 annually
- Annual endocrinology consultation: €150–250
- **Annual ongoing costs: €1,500–2,500 perpetually**

**Postoperative hematoma requiring reoperation:**

- Emergency operating theatre utilization: €1,500–2,500
- Surgical personnel time (re-exploration): €800–1,200
- Anesthetic time and consumables: €400–600
- Transfusion costs (if applicable): €200–500 per unit
- Extended hospital stay (median 1 additional day): €400–700
- Subtotal: €3,300–5,700 per event

**4. Activity-Based Costing Implementation**

**4.1 Cost Pool Definition and Allocation**

**Direct cost pools (unambiguous assignment to thyroidectomy pathway):**

1. Personnel costs for surgical team (surgeon, residents, nurses) - assigned 100% to procedure
2. IONM-specific consumables (electrodes, cables, recording media) - assigned 100% to procedure
3. Anesthetic drugs and monitoring consumables - assigned 100% to procedure
4. Postoperative medications (calcium, PTH, analgesics) - assigned 100% to procedure

**Indirect cost pools (allocated proportionally):**

1. Operating room overhead (facility rent, utilities, sterilization, housekeeping):
   - Annual facility cost ÷ annual operative hours per theatre × procedure time = €150–250 per case
2. Hospital administration and management:
   - Total annual administrative salary ÷ total annual hospital patient encounters × procedure weight (case complexity factor) = €50–100 per case
3. Equipment maintenance and biomedical support:
   - Annual maintenance contracts ÷ annual procedure volume per device = €40–80 per case for IONM systems

**4.2 Cost Allocation Methodology**

**Time-driven activity-based costing (TDABC):**

- Identify all activities in thyroidectomy pathway (preoperative consultation, operative setup, procedure, postoperative monitoring, follow-up)
- Measure time consumed by each activity (from structured interviews and EHR data)
- Calculate cost per minute for each resource (labor rate per minute)
- Multiply activity time × resource cost per minute = activity cost
- Aggregate activities across pathway = total episode cost

**Example calculation:**

- Preoperative surgeon consultation: 40 minutes × €50/minute = €2,000
- Operative time (surgeon): 120 minutes × €50/minute = €6,000
- Postoperative rounds and clinic: 50 minutes × €50/minute = €2,500
- **Total surgeon labor cost: €10,500 per case**

**4.3 Sensitivity Analysis on Cost Assumptions**

Cost estimates vary by:

- Geographic location (Europe vs. other regions)
- Healthcare system (public vs. private vs. mixed)
- Institution type (high-volume academic center vs. lower-volume community hospital)
- Surgeon experience and case complexity
- Complication rates and severity

**Scenario analyses:**

1. **Base case:** Medium-volume hospital, moderate complication rates, average equipment costs
2. **High-cost scenario:** Private institution, premium equipment, high staff salaries, above-average complication rates
3. **Low-cost scenario:** Lower-income region, refurbished equipment, lower labor costs
4. **High-risk case scenario:** Redo surgery, central compartment disease, higher complication probability

**5. Integration with Health Outcomes and Quality-Adjusted Life Year (QALY) Calculation**

**5.1 Health Outcome Measurement**

**Clinical outcomes captured across monitoring strategies:**

Nerve injury outcomes (primary focus):

- RLN paralysis incidence (transient vs. permanent)
- SLN injury with voice fatigue or reduced phonation range
- Combined bilateral RLN injury necessitating tracheostomy (rare but catastrophic)
- Vagal injury with dysphagia or aspiration risk

Metabolic complications:

- Hypoparathyroidism incidence and severity (permanent vs. transient)
- Serum calcium nadir and symptoms (numbness, tetany, seizures)
- Long-term requirement for calcium and calcitriol supplementation

Patient-reported outcomes:

- Voice quality (Voice Handicap Index–10, Acoustic voice measures)
- Swallowing function (Dysphagia Symptom Severity Scale)
- Neck pain or sensation disturbance
- Cosmetic satisfaction (appearance of surgical scar)
- Return to normal activities timeline

**5.2 QALY Derivation**

**Utility weights (EQ-5D-based or condition-specific instruments):**

Baseline health state (euthyroid, no complications): Utility = 1.0 (perfect health)

RLN palsy (transient, 3–6 months duration):

- Dysphonia and reduced voice projection: Utility decrement = 0.05–0.10
- Fatigue and communication difficulty: Additional decrement = 0.02–0.05
- **Total utility with transient RLN injury: 0.85–0.93**
- Duration: 6 months (0.5 years)
- QALYs lost = (1.0 – 0.90) × 0.5 = 0.05 QALYs

RLN palsy (permanent):

- Persistent dysphonia, reduced vocal endurance: Utility = 0.80–0.85
- Restriction on occupation if voice-dependent profession
- QALYs lost over lifetime (assuming 30-year horizon post-surgery) = (1.0 – 0.82) × 30 = 5.4 QALYs

Transient hypoparathyroidism (3–6 months):

- Symptoms (paresthesia, tetany if severe): Utility = 0.92–0.97
- Treatment burden (daily medications, frequent lab tests): Utility decrement = 0.02–0.05
- **Total utility: 0.93–0.95**
- QALYs lost = (1.0 – 0.94) × 0.5 = 0.03 QALYs

Permanent hypoparathyroidism:

- Long-term supplementation and monitoring: Utility = 0.95–0.98 (minimal impact for most patients)
- QALYs lost (30-year horizon) = (1.0 – 0.96) × 30 = 1.2 QALYs

**5.3 Cost-Effectiveness Calculation**

**Incremental cost-utility ratio (ICUR):**

ICUR = (Cost_CIONM – Cost_Visual) / (QALYs_CIONM – QALYs_Visual)

**Example scenario (per 100 thyroidectomy cases):**

Visual identification alone:

- Total cost: 100 cases × €1,300 = €130,000
- RLN injury rate: 2.0% (2 permanent injuries)
- Hypoparathyroidism rate: 1.5% permanent
- QALYs lost: (2 × 5.4) + (1.5 × 1.2) = 10.8 + 1.8 = 12.6 QALYs lost
- Net cost per 100 patients: €130,000 + (2 × €15,000 for vocal cord surgery) + (1.5 × €1,500/year × 30 years = €67,500) = €212,500

CIONM:

- Total cost: 100 cases × €2,000 = €200,000
- RLN injury rate: 0.5% (0.5 permanent injuries, 1.0% transient)
- Hypoparathyroidism rate: 0.5% permanent
- QALYs lost: (0.5 × 5.4) + (1.0 × 0.05) + (0.5 × 1.2) = 2.7 + 0.05 + 0.6 = 3.35 QALYs lost
- Net cost per 100 patients: €200,000 + (0.5 × €15,000) + (0.5 × €67,500/30 years × 30) = €200,000 + €7,500 + €1,125 = €208,625

**CIONM is MORE expensive but also prevents QALYs from being lost**

**Recalculating as net benefit framework:**

- Cost difference: €208,625 – €212,500 = –€3,875 (CIONM actually saves money)
- QALY difference: 12.6 – 3.35 = 9.25 QALYs gained with CIONM
- ICUR = –€3,875 / 9.25 = **Dominant: CIONM is less costly AND more effective (dominance)**

This example demonstrates how CIONM, despite higher device costs, may be economically dominant when comprehensive complication prevention is considered.
