# Supplementary material for: Value‐Based Neuromonitoring in Thyroidectomy: A Comprehensive Cost–Utility Analysis
Source: Laryngoscope. 2026 Mar 31;136(7):3271–83. doi: 10.1002/lary.70536 (PMC13253177; doi:10.1002/lary.70536)
Supplement: Supplementary file 4 — Data S3: Study arms and analyses. [file LARY-136-3271-s004.docx]

**Supplementary Material 4. Study arms and analyses**

| **Domain** | **Category** | **Definition / Description** |
| --- | --- | --- |
| ***Study arms*** | No neuromonitoring | RLN management based solely on visual identification without electrophysiologic monitoring. |
|  | Laryngeal palpation | Manual palpation of the larynx to confirm vocal cord contraction after RLN dissection and at predefined intraoperative milestones. |
|  | Needle-based IONM | Intermittent monitoring using percutaneous or transcartilaginous needle electrodes placed in the laryngeal musculature (e.g., cricothyroid muscle). |
|  | Intermittent IONM | Standardized stimulation and EMG recording of RLN and/or vagus nerve at key steps, with analyses stratified by monitoring system manufacturer. |
|  | Continuous IONM (CIONM) | Real-time surveillance of vagus or RLN function via continuous stimulation and EMG recording, stratified by device manufacturer. |
|  | Automated trend monitoring | Automated nerve trend–monitoring platforms (Medtronic) using continuous EMG analysis and predefined alarm thresholds for early detection of signal deterioration. |
| ***Subgroup analyses*** | Electrode configuration | Integrated EMG endotracheal tube versus stitched-on or surface laryngeal electrodes. |
|  | Surgical volume | High- versus low-volume surgeons and institutions, classified according to annual thyroidectomy case load. |
|  | RLN injury risk | Prespecified proportional reduction in RLN injury rates associated with each monitoring strategy, derived from pooled contemporary series. |
| ***Outcomes – primary*** | Neuromonitoring cost | Total neuromonitoring-related costs, including capital equipment, disposable supplies, maintenance, and training. ​ |
|  | Net cost offset | Difference in RLN injury–related costs between monitored and unmonitored strategies, expressed as net cost savings or excess cost. |
|  | Laryngoscopy use | Use of routine versus selective preoperative and postoperative laryngoscopy to document vocal cord mobility. |
|  | Cost per patient | Direct and indirect per-patient costs (hospitalisation, rehabilitation, medicolegal expenditures, productivity losses). |
|  | QALYs | Quality-adjusted life years lost or gained for each neuromonitoring strategy. |
|  | Technical performance | Incidence, type, and pattern of neuromonitoring technical failures or signal loss. ​ |
| ***Manufacturer analyses*** | Medtronic | NIM platforms (e.g., NIM-Response, NIM-Neuro, NIM Vital) supporting intermittent IONM and CIONM with EMG tubes and APS vagus electrodes, including NerveTrend automated EMG trend analysis. |
|  | Inomed | C2 and C2 Xplore systems for cranial nerve monitoring with thyroid/ENT modules, enabling intermittent and continuous RLN monitoring with standardized probes and multichannel EMG. |
|  | Dr. Langer Medical | AVALANCHE SI 2 and related EMG platforms tailored to endocrine neck surgery, providing intermittent IONM with procedure-specific stimulation and recording configurations. |
|  | NCC Medical | NCC Smart IONM, a multipurpose neuromonitoring console with configurable channels used for RLN monitoring during thyroidectomy. |
|  | Natus | General-purpose intraoperative neuromonitoring systems included when employed for RLN monitoring in thyroid surgery, reflecting heterogeneous device availability. |
|  | Neurovision | “Universal” EMG laryngeal electrodes and EMG endotracheal tubes compatible with multiple consoles, representing a major component of the disposable electrode market and enabling cross-platform recording standardization. |
| ***Statistical methods*** | Clinical endpoints | Univariable and multivariable analyses of RLN injury and other categorical outcomes using chi-square or Fisher exact tests and multivariable logistic regression to estimate adjusted odds ratios and 95% confidence intervals. |
|  | Continuous endpoints | Analysis of cost per case and operative time using ANOVA or linear regression, with generalized linear models or non-parametric tests for skewed or heteroscedastic distributions. |
| ***Economic evaluation*** | Cost-effectiveness | Incremental cost-effectiveness ratios calculated as differences in mean costs divided by differences in clinical benefit (RLN injuries averted or QALYs gained). |
|  | Uncertainty analysis | Non-parametric bootstrapping to generate cost–effectiveness planes and acceptability curves; regression-based incremental net benefit as an alternative summary measure. |
| ***Sensitivity analyses*** | Deterministic | One-way and multi-way sensitivity analyses varying surgical volume, technology mix, unit costs, and hospital setting (academic vs community), plus scenario analyses for RLN injury rates, medicolegal costs, and laryngoscopy strategies. |
|  | Probabilistic | Probabilistic sensitivity analysis with parameter-specific distributions to characterise joint uncertainty in costs and outcomes. |
| ***DRG comparison*** | Tariff alignment | Comparison of total hospitalization and procedure costs under each monitoring scenario with the Italian DRG 290 reimbursement tariff for thyroid surgery, stratified by monitoring strategy and volume category to identify funding gaps and budget impact. |
